# Emodin Suppresses the RAGE/ROCK1 Pathway to Modulate the Oligodendrocyte Lineage and Ameliorate Demyelination in Vascular Dementia Rats

**DOI:** 10.1002/cns.71154

**Published:** 2026-09-16

**Authors:** Xueqing Wang, Xiumei Dong, Cunbao He, Junjie Zhang, Wenming Ban, Guoqi Zhu, Jingji Wang, Yilan Zhen

**Affiliations:** ^1^ Key Laboratory of Xin'an Medicine, the Ministry of Education Anhui University of Chinese Medicine Hefei China; ^2^ Department of Pharmacy, the First Affiliated Hospital of USTC, Division of Life Sciences and Medicine University of Science and Technology of China Hefei China; ^3^ Taihe Hospital of Traditional Chinese Medicine Anhui University of Traditional Chinese Medicine Fuyang China; ^4^ Key Laboratory of Molecular Biology (Brain Disease) Anhui University of Chinese Medicine Hefei China; ^5^ The Second Affiliation Hospital of Anhui University of Chinese Medicine Hefei China; ^6^ College of Integrated Chinese and Western Medicine Anhui University of Chinese Medicine Hefei China

**Keywords:** demyelination, emodin, oligodendrocytes, remyelination, vascular dementia

## Abstract

**Background:**

Vascular dementia (VD), a prevalent cognitive disorder mainly resulting from chronic cerebral hypoperfusion‐triggered white matter injury (WMI), takes regulation of oligodendrocyte (OL) lineage and restoration of remyelination as key therapeutic targets, whereas the precise role and mechanism of the natural neuroprotective compound emodin in VD‐related demyelination have yet to be elucidated.

**Methods:**

A VD rat model was generated by bilateral common carotid artery occlusion. After 2 weeks of emodin administration, cognitive function, myelination, and OL lineage were evaluated using behavioral tests, histology, electron microscopy, and immunofluorescence. A comprehensive approach combining proteomics, network pharmacology, molecular docking, dynamics simulations, cellular thermal shift assay (CETSA), and both pharmacological inhibition and activation was applied to systematically elucidate the underlying mechanism of emodin.

**Results:**

Emodin administration significantly improved cognitive deficits and reduced WMI in VD rats. It effectively modulated the OL lineage by reducing the elevated proliferative response of OPCs while robustly promoting their maturation and remyelination and inhibiting the RAGE/ROCK1 signaling pathway in the corpus callosum and hippocampus of VD rats. Mechanistically, CETSA revealed engagement between emodin and RAGE/ROCK1, and pharmacological inhibition of either RAGE or ROCK1 mimicked the therapeutic benefits of emodin. Conversely, ROCK1 activation reversed emodin‐induced improvements in OL maturation.

**Conclusion:**

Our study reveals that emodin modulates the OL lineage to promote remyelination in VD by suppressing the RAGE/ROCK1 pathway. These results suggest a potential molecular mechanism for emodin and highlight the RAGE/ROCK1 axis as a therapeutic target for VD.

## Graphical abstract

Emodin improves white matter remyelination and alleviates cognitive deficits through suppression of the RAGE/ROCK1 signaling pathway in VD rats. Mechanistically, it modulates the OL lineage by reducing the elevated proliferative response of OPCs while promoting their maturation and remyelination.

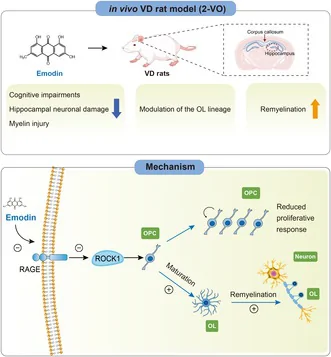

**Abbreviations**
CC: corpus callosum
CETSA: cellular thermal shift assay
DG: dentate gyrus
HIP: hippocampus
IC: internal capsule
LFB: Luxol Fast Blue
MBP: myelin basic protein
MOG: myelin oligodendrocyte glycoprotein
MWM: Morris water maze
NF200: Neurofilament Protein 200
NOR: new object recognition
OL: oligodendrocyte
OPC: oligodendrocyte progenitor cell
TEM: Transmission Electron Microscopy
VD: vascular dementia

## 1 Introduction

Vascular dementia (VD) involves complex pathophysiology, with chronic cerebral hypoperfusion (CCH) recognized as a key contributor, particularly through its impact on white matter [1]. White matter injury (WMI), characterized by oligodendrocyte (OL) loss and subsequent demyelination, is a major pathological substrate for cognitive decline in VD [2]. In response to demyelination, oligodendrocyte precursor cells (OPCs) are attracted to the injury site, where they expand and migrate [3]. These OPCs further differentiate into mature OLs, which remyelinate axons by forming new myelin sheaths [4]. This regenerative process not only restores neuronal signaling efficiency but also protects nerve fibers from additional injury [5]. Given that alterations in OL lineage cells directly compromise myelin integrity [6], promoting OPC differentiation, maturation, and remyelination represents a promising therapeutic strategy for WMI. Nevertheless, the exact mechanisms driving VD onset, the extent of myelin disruption, and potential therapeutic targets remain to be fully elucidated.

Pharmacological agents that promote remyelination emerge as a promising strategy for developing effective treatments against VD [1]. Emodin (1,3,8‐trihydroxy‐6‐methylanthraquinone), a natural anthraquinone derived from rhubarb and other traditional Chinese medicinal herbs, exhibits a range of pharmacological activities, including antioxidant, anticancer, and antitumor effects [7, 8, 9]. Furthermore, emodin inhibits amyloid‐β (Aβ) aggregation and mitigates its associated cytotoxicity in Alzheimer's disease models [10]. It suppresses reactive oxygen species (ROS) overproduction in cerebral ischemia [11], depletes glutathione [12], and inhibits caspase‐3 activation [13]. Emodin reduces oxidative stress and alleviates synaptic dysfunction in SH‐SY5Y neuroblastoma cells [14, 15], thus providing neuroprotection. In a recent experimental autoimmune encephalomyelitis (EAE) model, emodin could block microglial activation, confer anti‐inflammatory and antioxidant effects, and ameliorate the hypoxic–ischemic milieu, thereby maintaining normal myelin function [16, 17]. These evidences underscore its potential in remyelination. However, the role and underlying mechanisms of emodin in VD‐related cerebral WMI and cognitive dysfunction remain to be elucidated.

To further investigate the regulatory effects of emodin on the OL lineage, this study examines the alterations in proliferation, differentiation, and maturation of OLs in the corpus callosum (CC) and hippocampus of VD rats following emodin treatment, and explores how this process contributes to the mechanism by which emodin alleviates myelin lesions in VD. Our research clarifies the specific signaling mechanisms through which emodin regulates the OL lineage and myelin repair, providing novel theoretical insights and identifying potential therapeutic approaches for preventing and treating VD.

## 2 Materials and Methods

### 2.1 Animals and Groups

Male Sprague–Dawley (SD) rats, weighing around 250 g and aged between 6 to 8 weeks, were supplied by Liaoning Changsheng Biotechnology Co. Ltd. (license: SCXK[Liao]2020‐0001). Rats were housed under controlled temperature (20°C–26°C) and humidity (40%–70%) with free access to food and water. They were acclimated to 12‐h light/dark conditions for 7 days before being randomly assigned to various experimental groups using a random number table.

Male SD rats were distributed randomly into the following six groups (*n* = 12 each): sham‐control, VD model, three groups receiving emodin (10, 20, or 40 mg/kg, *i.p*. [16, 18]), and an oxiracetam positive control group (50 mg/kg, *i.p*. [19]). Saline was administered intraperitoneally to the sham and VD model animals. Emodin (95% purity, S30748) and oxiracetam (H20030037) were purchased from Shanghai Yuanye Bio‐Technology Co. Ltd. and Hunan Jianlang Pharmaceutical Co. Ltd., China, respectively.

For inhibitor validation, male SD rats comprised sham‐control, VD model, VD treated with FPS‐ZM1 (1 mg/kg, *i.p*., HY‐19370; MCE, USA) [20], and VD treated with Fasudil (10 mg/kg, *i.p*., HY‐10341A, MCE, USA) [21] groups (*n* = 9 each). Treatments were given daily for 2 weeks, with control and VD groups receiving saline of the same volume.

For agonist validation, male SD rats were divided into VD groups treated with emodin (40 mg/kg, *i.p*.), U46619 (30 μg/kg, *i.p*., S28454, Yuanye, Shanghai) [22], or a combination of U46619 and emodin (*n* = 10 each). Treatments were given daily for 2 weeks.

Behavioral tests, histological, and immunofluorescence quantifications were performed by investigators blinded to group allocation.

### 2.2 Animal Model Preparation

As we previously described, a rat model for bilateral common carotid artery occlusion (2‐VO) was generated [23]. Briefly, under isoflurane anesthesia (flow rate 0.41 mL/min, maintenance concentration 2%), the left common carotid artery was surgically isolated via a midline cervical incision and permanently ligated. After intramuscular injection of ampicillin (0.1 mL) and disinfection with iodophor, the wound was sutured. The same procedure was repeated on the right carotid artery 1 week later. During the interval between the two surgeries, the mortality rate was 2%. Sham‐operated rats received exposure only. All pharmacological treatments were initiated on the 21st day following the completion of bilateral occlusion.

### 2.3 Novel Object Recognition (NOR)

The NOR test was performed as previously described [24]. Briefly, the equipment consisted of a 90‐cm cubic open field. Each rat was first put in the field for 5 min to acclimate. On the following day, rats explored two identical objects until accumulating 20 s of investigation. After 24 h, one object was replaced with a novel one, and exploration was recorded over 5 min. Cognitive performance was compared using the recognition index (RI = TN/[TN + TF]), where TN and TF represent time spent with the novel and familiar objects, respectively.

### 2.4 Morris Water Maze (MWM)

Spatial learning and memory were subsequently assessed in the MWM, with testing conducted over 5 days following the NOR. Prior to formal trials, rats were allowed a 2‐min free swim to minimize stress. During acquisition trials, rats were sequentially released from the four quadrants to locate a submerged hidden platform within 90 s. Escape latency was recorded; rats failing to find the platform within the time limit were guided to it and allowed to remain for 15 s. On the final day, a spatial exploration test was conducted with the platform removed, during which the duration spent and platform crossings in the target quadrant were quantified over 90 s.

### 2.5 Sample Processing

Following behavioral tests, rats were sacrificed under isoflurane anesthesia followed by decapitation. Blood was obtained through abdominal aortic puncture for subsequent serum isolation. Brains were rapidly removed; the hippocampus and CC were dissected on ice for electron microscopy, western blot, proteomics, and RT‐PCR analyses. For hematoxylin–eosin, luxol fast blue and immunofluorescence staining, brains were fixed in paraformaldehyde prior to embedding, sectioning, and staining.

### 2.6 HE Staining

Each group of rats was euthanized via neck dislocation, and brain tissues were immediately collected. Following fixation, dehydration, and embedding, 4‐μm paraffin sections were prepared, with three randomly selected sections per specimen. Hippocampal damage was then assessed via hematoxylin and eosin staining [23]. Histopathological assessment of the hippocampus was performed using light microscopy (Olympus, Japan).

### 2.7 ELISA Assay

Commercial kits (NJJCBIO, C010‐2‐1 for AST/GOT and C009‐2‐1 for ALT/GPT), BUN test kit (NJJCBIO, C013‐2‐1), and CRE kit (NJJCBIO, C011‐2‐1) were used to assess the enzymatic activities of AST/GOT and ALT/GPT, blood urea nitrogen (BUN), and creatinine (CRE) following the instructions of the assay kits.

### 2.8 Transmission Electron Microscopy (TEM)

Brain tissue samples (1 mm × 1 mm × 1 mm) from the CC and hippocampus were fixed in 2.5% glutaraldehyde and embedded for ultramicrotomy. Ultrathin sections (70 nm) were prepared using a Leica UC7, double‐stained with uranyl acetate and lead citrate, and observed under a Hitachi 7700 TEM. Myelin integrity was quantitatively assessed by calculating the G‐ratio (inner axonal to total fiber diameter).

### 2.9 Immunofluorescence Staining

Following fixation in 4% paraformaldehyde and cryoprotection in 30% sucrose, brain tissues were sectioned at 35 μm. For immunofluorescence, sections underwent blocking in PBS containing 10% goat serum and 0.4% Triton X‐100 as previously described [25]. Primary antibodies applied overnight at 4°C included: Ki67 (1:100, ABclonal, A23722), NG2 (1:50, Merck, 4,146,505), CC1 (1:100, Millipore, OP80), OLIG2 (1:200, Cell Signaling Technology, 65915S), myelin basic protein (MBP, 1:200, Boster, BAO094), and neurofilament 200 (NF200, 1:200, Boster, BM0100). After PBS washes, sections were treated with fluorescent secondary antibodies (FITC‐anti‐rabbit or Alexa Fluor 594‐anti‐mouse; 1:100; ZS‐Bio) for 2 h, stained with DAPI (Beyotime, C1005), and visualized using an Olympus FV1000 confocal microscope. In the hippocampus and CC, the proportions of CC1^+^OLIG2^+^ cells among OLIG2^+^ cells and of Ki67^+^NG2^+^ cells among NG2^+^ cells, along with the fluorescence intensities of MBP and NF200, were quantified using ImageJ. These analyses assessed oligodendrocyte proliferation, maturation, and myelination in the VD model.

### 2.10 Western Blotting

Tissue samples from the CC and hippocampus were homogenized in lysis buffer. Following protein quantification, equal amounts were subjected to SDS‐PAGE, followed by transfer to PVDF membranes. The proteins were incubated with primary antibodies targeting MBP (1:1000, Boster, BAO094), MOG (1:1000, CST, 45268S), ROCK1 (1:1000, BOSTER, BM4203), RAGE (1:1000, Selleck, A5699), and β‐actin (1:1000, ZENBIO, 200068‐8F10) overnight at 4°C. After washing and incubation with secondary antibodies, signal detection was carried out with ECL substrate on a ProteinSimple imaging system (USA), and band intensities were quantified with ImageJ.

### 2.11 Real‐Time qPCR

Total RNA was extracted from CC tissue using TRNzol reagent (Tiangen, DP424). Following homogenization, samples were chloroform‐extracted (Sinopharm, 10006818) and centrifuged for phase separation. The aqueous phase was collected, mixed with chilled isopropanol (Sinopharm, 80109218) for RNA precipitation at −20°C. The RNA precipitate was resuspended in 75% ethanol to eliminate residual salts and genomic DNA contamination. Purified RNA was reverse transcribed into cDNA. The ABI Step One Real‐Time PCR system was utilized for conducting qPCR. Target gene Ct values were calculated and assessed using the 2^−△△Ct^ approach. Gene primer sequences used in this study are outlined in Table 1.

**TABLE 1:** Primer names and sequences.

Primer name	Primer sequence (5′–3′)	
*ROCK1*	Forward	AAACTTTTGGACCTTTCGGATTC
	Reverse	TTTTGCTGCTCACCACAACATAC
*Vamp2*	Forward	TGAAACAAGTGCAGCCAAGC
	Reverse	GCGCAAATCACTCCCAAGATG
*Acap2*	Forward	GAGCCAGCAACGCCTTCAAAAC
	Reverse	GAGACGACCTCAAAGCAGAACCG
*Jmy*	Forward	ATGTGGACGGTGCTGTTTGG
	Reverse	GCTCGCTGAGGCTTTCGTAAT
*PCDH7*	Forward	GTGGGAGCAGGAGACAACATTTC
	Reverse	AGCCCGCTGTCATAGCAACTC
*GPC5*	Forward	AGATTGCCGCTCAGCAGGATTT
	Reverse	TCGGATGAGGGTTTCCAGGGTC
*β‐Actin*	Forward	GTCGTACCACTGGCATTGTG
	Reverse	TCTCAGCTGTGGTGGTGAAG

### 2.12 Luxol Fast Blue (LFB) Staining

Brain sections were dewaxed and incubated with Myelin Stain A at 60°C for 30 min, followed by a one‐hour incubation under coverslips. After rinsing, sections were briefly immersed in Myelin Dye B (2 s) and then in Myelin Dye C (15 s). Following thorough washing, sections were examined microscopically until achieving distinct blue myelin sheaths against near‐colorless backgrounds. Sections were then dried at 65°C for approximately 30 min, cooled in 95% ethanol, counterstained with eosin, and mounted with neutral gum. Images were acquired and analyzed using standard fluorescence microscopy.

### 2.13 Proteomics Analysis and Bioinformatic Screening of Candidate Targets

Hippocampal tissues were collected from VD model and control rats (*n* = 4 per group) to identify myelin repair‐related targets. Proteins were extracted, digested, and analyzed by LC–MS/MS as previously described [26]. Differentially expressed proteins (|Log2FC| ≥ 1.2, *p* < 0.05) underwent functional enrichment analysis, including Gene Ontology (GO) terms and Kyoto Encyclopedia of Genes and Genomes (KEGG) pathways, via the DAVID online platform. Candidate proteins were selected with a focus on biological processes related to OL differentiation, maturation, and myelination, established signaling pathways regulating oligodendrocyte development and myelination, and pathways associated with neurological disorders involving OL dysfunction. Application of these criteria to the enrichment results identified the final candidate targets for experimental validation.

### 2.14 Molecular Docking

The emodin sdf file, which includes its 3D and 2D structures, was retrieved from PubChem and subsequently processed into mol2 format via the Open Babel graphical user interface. Protein identifiers for NOTCH1, RAF, RAGE, SIRT1, and RHOA were collected from Uniprot, while PDB files were sourced from the Protein Data Bank. The structures of RAS proteins were constructed using SWISS‐Model. Docking simulations of emodin with the receptors NOTCH1, RAF, RAGE, SIRT1, RHOA, and ROCK1 were performed using PyMOL version 3.7 in conjunction with AutoDock Tools 1.5.7. Binding energies below −5.0 kcal/mol were considered favorable, and those under −9.0 kcal/mol indicated strong affinity. Interaction sites and bond lengths were rendered using PyMOL 2.6.

### 2.15 Molecular Dynamics

All molecular dynamics simulations were conducted with the Amber ff14SB force field and TIP3P water model using Amber24 software. The protein–ligand system was solvated in a cubic water box. Electrostatic and van der Waals interactions were treated with a 1.0‐nm cutoff, and a time step of 2 fs was used. The Particle Mesh Ewald (PME) method was applied to model long‐range electrostatics. After initial energy minimization, sequential equilibration was conducted under NVE (200 ps) and NPT (100 ps) ensembles at 300 K and 1 atm. Temperature and pressure were regulated using the V‐rescale and Parrinello–Rahman methods, respectively. A 100‐ns production run was carried out, during which Cpptraj and Python were utilized to derive RMSD, RMSF, radius of gyration, and binding free energy.

### 2.16 Cellular Thermal Shift Assay (CETSA)

For thermal stability assessment, CC tissues from the model group and the emodin‐treated group were separately homogenized and resuspended in PBS to prepare single‐cell suspensions. After lysis, each lysate was equally aliquoted into six portions and then heated at 40°C, 46°C, 52°C, 58°C, 64°C, and 70°C for 3 min, respectively. Following centrifugation, supernatants were collected for western blotting to determine the expression levels of target proteins.

### 2.17 Statistical Analyses

Data analysis utilized GraphPad Prism version 9.0, with findings presented as Means ± SEM. Normality was verified using the Shapiro–Wilk test. For multi‐group comparisons, one‐way ANOVA followed by Tukey's test was used for normally distributed data; otherwise, the Kruskal–Wallis test was employed. Significance was set at *p* < 0.05.

## 3 Results

### 3.1 Emodin Attenuates Cognitive Dysfunction and Hippocampal Neuronal Damage in VD Rats

**FIGURE 1:**
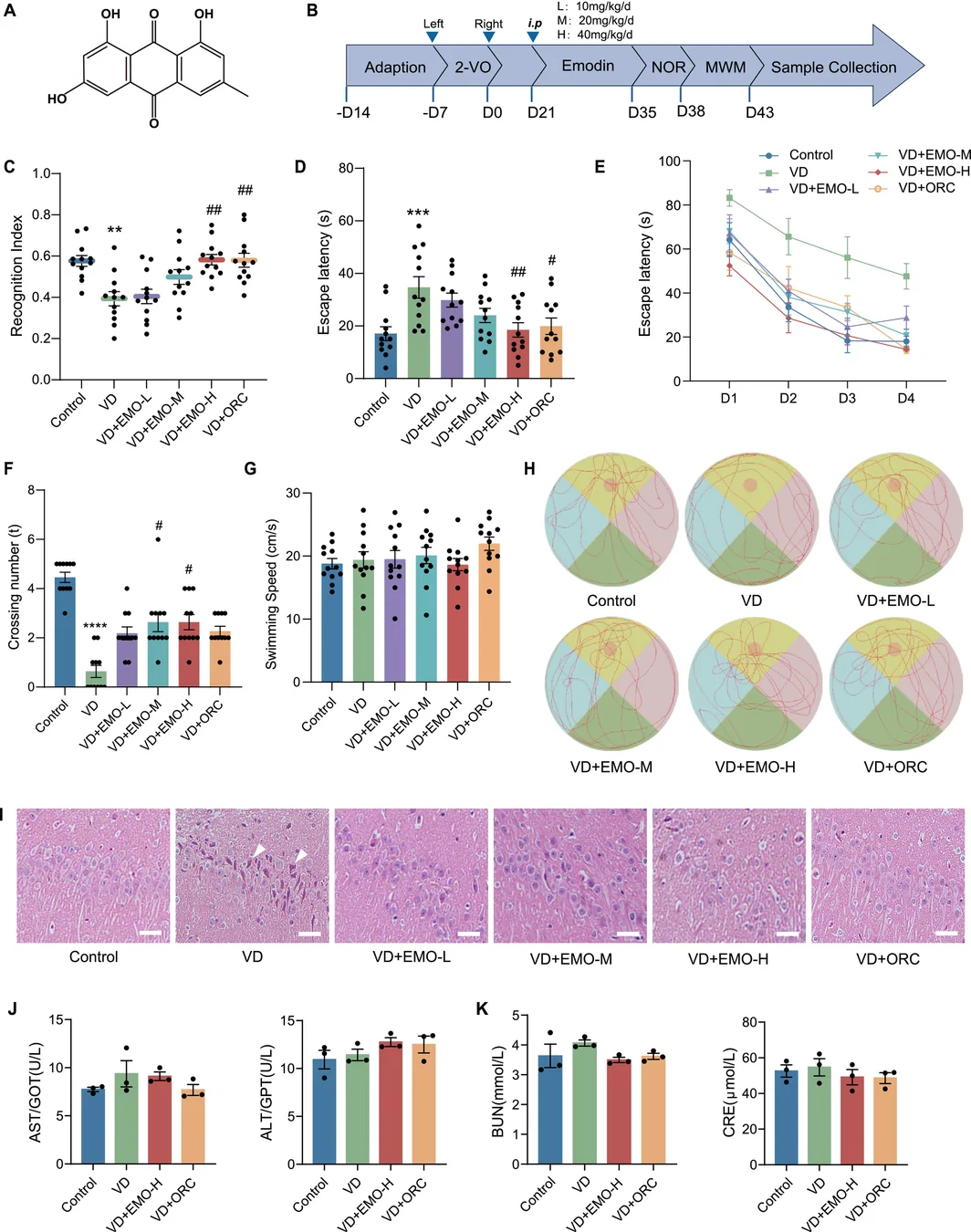
Emodin attenuates cognitive dysfunction and hippocampal neuronal damage in VD rats. (A) Chemical structure of emodin. (B) Experimental timeline for in vivo studies. Left indicates left common carotid artery ligation, right indicates right common carotid artery ligation, H, high‐dose emodin; L, low‐dose emodin; M, medium‐dose emodin. (C) NOR cognitive index across groups (*n* = 12). (D) Escape latency, (E) average escape latency for the first 4 training days, (F) number of platform crossings, and (G) swimming speed in the MWM (*n* = 12 per group). (H) Representative search trajectories of each group in the MWM. (I) Histological morphology of hippocampal CA1 neurons. White arrows indicate abnormal neurons (HE staining, 10×, scale bar: 20 μm). (J) Evaluation of liver injury in each group (*n* = 3). (K) Evaluation of kidney injury in VD rats in each group (*n* = 3). (Control: Control group, VD: 2‐VO model group, VD + EMO‐L: 2‐VO + low‐dose emodin group, VD + EMO‐M: 2‐VO + medium‐dose emodin group, VD + EMO‐H: 2‐VO + high‐dose emodin group, VD + ORC: 2‐VO + oxiracetam group. **p < 0.01, ***p < 0.001, ****p < 0.0001 versus Control; #*p* < 0.05, ##*p* < 0.01 versus VD; data are represented as mean ± SEM).

To evaluate the therapeutic effects of emodin, a VD model was first created in rats using 2‐VO surgery followed by emodin administration. Cognitive assessments, including the MWM and NOR tests, were then conducted (Figure 1A,B). Compared with controls, VD rats exhibited a lower cognitive index (Figure 1C), significantly longer escape latency (Figure 1D), and higher average escape latency for the first four training days (Figure 1E), along with fewer platform crossings (Figure 1F,H). Swimming speed did not differ significantly between groups (Figure 1G). Notably, the cognitive index was considerably enhanced by high‐dose emodin and oxiracetam treatments in comparison to the VD rats (Figure 1C). All emodin‐treated groups showed reduced escape latency during the initial four‐day periods, with the greatest improvement observed in the high‐dose group (Figure 1D,E). Moreover, the groups receiving medium and high doses of emodin exhibited a significantly greater number of platform crossings than the VD group (Figure 1F,H).

HE staining showed hippocampal CA1 neuronal injury severity after emodin treatment (Figure 1I). Control neurons were densely arranged, with intact cell membranes and clear nuclei. Conversely, the VD group exhibited neuron loss and shrunken nuclei. Administration of oxiracetam or any dose of emodin alleviated neuronal damage and improved neuronal organization. In particular, high‐dose emodin provided the most significant neuroprotection relative to the VD model.

Quantification was performed on serum biomarkers of hepatic (AST/GOT, ALT/GPT) and renal (BUN, CRE) function in the VD group. Levels of these metabolites in the VD group, high‐dose emodin group, and oxiracetam group all fell within normal physiological ranges and displayed no notable differences from the control group (Figure 1J,K).

### 3.2 Emodin Reduces Myelin Injury and Promotes Remyelination in the CC and Hippocampus of VD Rats

**FIGURE 2:**
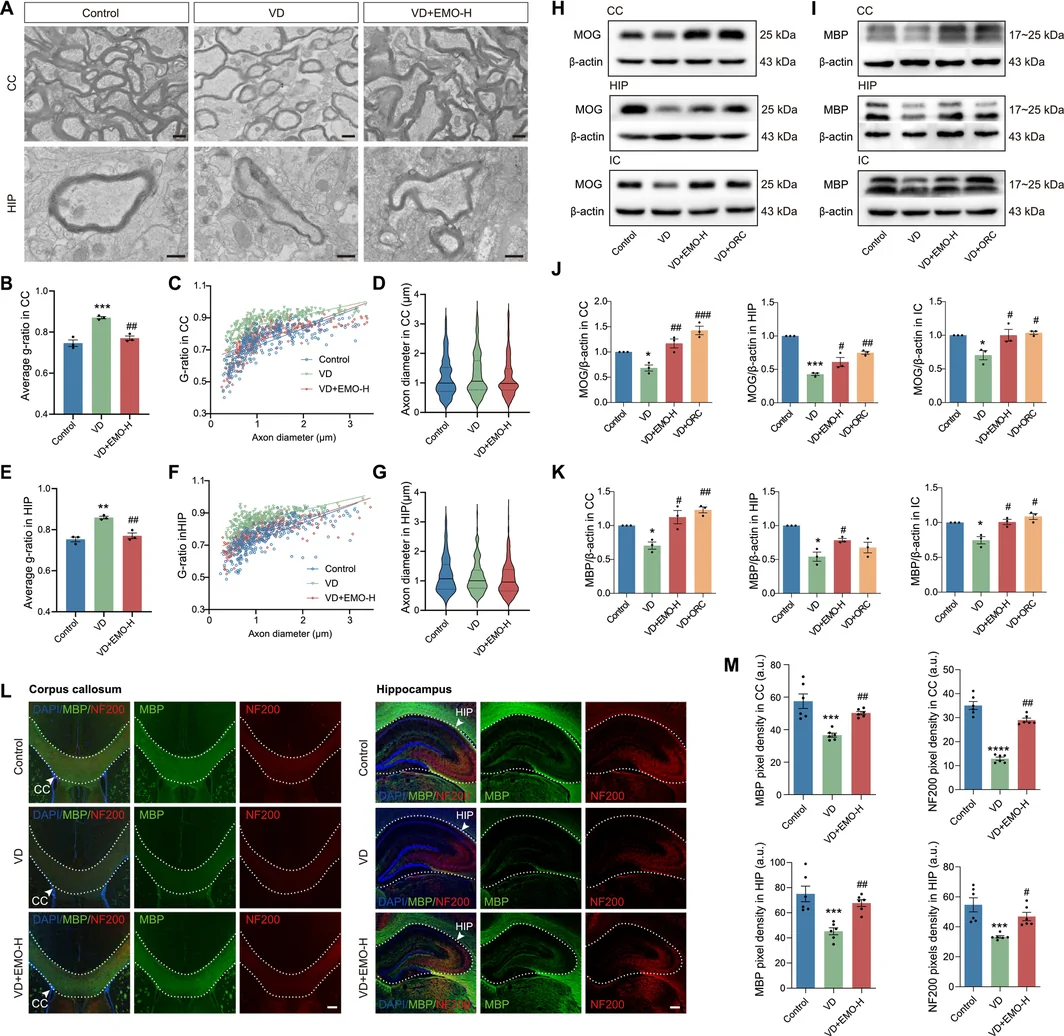
Emodin promotes remyelination in rats with VD. (A) TEM images of myelin in the CC and HIP of each group. Scale bar: Cc, 12 μm; HIP, 0.6 μm. (B) Average g‐ratio, (C) g‐ratio versus axon diameter scatter plots, and (D) axon diameter for the control, VD, and VD + EMO‐H groups in the CC. *n* = 304, 304, and 309 axons (3 rats/group), respectively. (E) Average g‐ratio, (F) g‐ratio versus axon diameter scatter plots, and (G) axon diameter for the control, VD, and VD + EMO‐H groups in the HIP. *n* = 306, 305, and 307 axons (3 rats/group), respectively. (H, I) Western blot images of MOG and MBP Expression in the CC, HIP and IC. (J, K) Statistical analysis of MOG and MBP expression (normalized to β‐Actin, *n* = 3). (L) Immunostaining of MBP (green) and NF200 (red) in CC and HIP. White dashed lines denote the CC and HIP borders. Scale bar: 200 μm. (M) Immunofluorescence quantification of MBP and NF200 in the CC and HIP, with *n* = 6 per group (*p < 0.05, **p < 0.01, ***p < 0.001, ****p < 0.0001 vs. Control; #*p* < 0.05, ##*p* < 0.01, ###*p* < 0.001 vs. VD; data are represented as mean ± SEM; CC, corpus callosum; HIP, hippocampus; IC, internal capsule).

To assess emodin's effect on myelin integrity in VD rats, transmission TEM was used to examine myelin ultrastructure. TEM revealed marked myelin disorganization, including loose packing, widened interlamellar gaps, and irregular sheath outlines in the CC and hippocampus of VD rats, contrasting with the tightly packed myelin sheaths in control animals. Treatment with high‐dose emodin effectively restored the myelin organization (Figure 2A). The g‐ratio, a key metric for assessing myelin thickness, reflects the degree of myelination, where lower values indicate thicker myelin sheaths [26]. Accordingly, g‐ratio analysis confirmed significant myelin thinning in VD rats both in CC and hippocampus, which was ameliorated by high‐dose emodin (Figure 2B–G). Furthermore, apoptosis within the CC was evaluated using cleaved caspase‐3 as a marker [27]. VD‐induced apoptosis in the CC, indicated by elevated cleaved caspase‐3 fluorescence intensity, was significantly reduced by emodin treatment (Figure S1).

The myelin proteins MOG and MBP are essential for maintaining the structural integrity and function of myelin sheaths [28]. To investigate the potential of emodin in promoting remyelination in VD rats, the expression of MOG and MBP was examined in the CC, hippocampus, and internal capsule (IC). As illustrated in Figure 2H–K, MOG and MBP expression levels were significantly reduced in VD rats across all three regions compared to the control rats. Both high‐dose emodin and oxiracetam treatments substantially restored MOG and MBP levels. CC MBP levels correlated negatively with MWM escape latency and positively with NOR recognition index (Figure S2). Immunofluorescence staining further corroborated these findings, demonstrating that the reduced fluorescence intensities of MBP and the axonal marker NF200 both in the CC and hippocampus of VD rats were markedly elevated following high‐dose emodin administration (Figure 2L,M). Collectively, these results indicate that emodin promotes remyelination and axonal preservation in VD.

### 3.3 Emodin Regulates the OL Lineage in the CC and Dentate Gyrus of VD Rats

**FIGURE 3:**
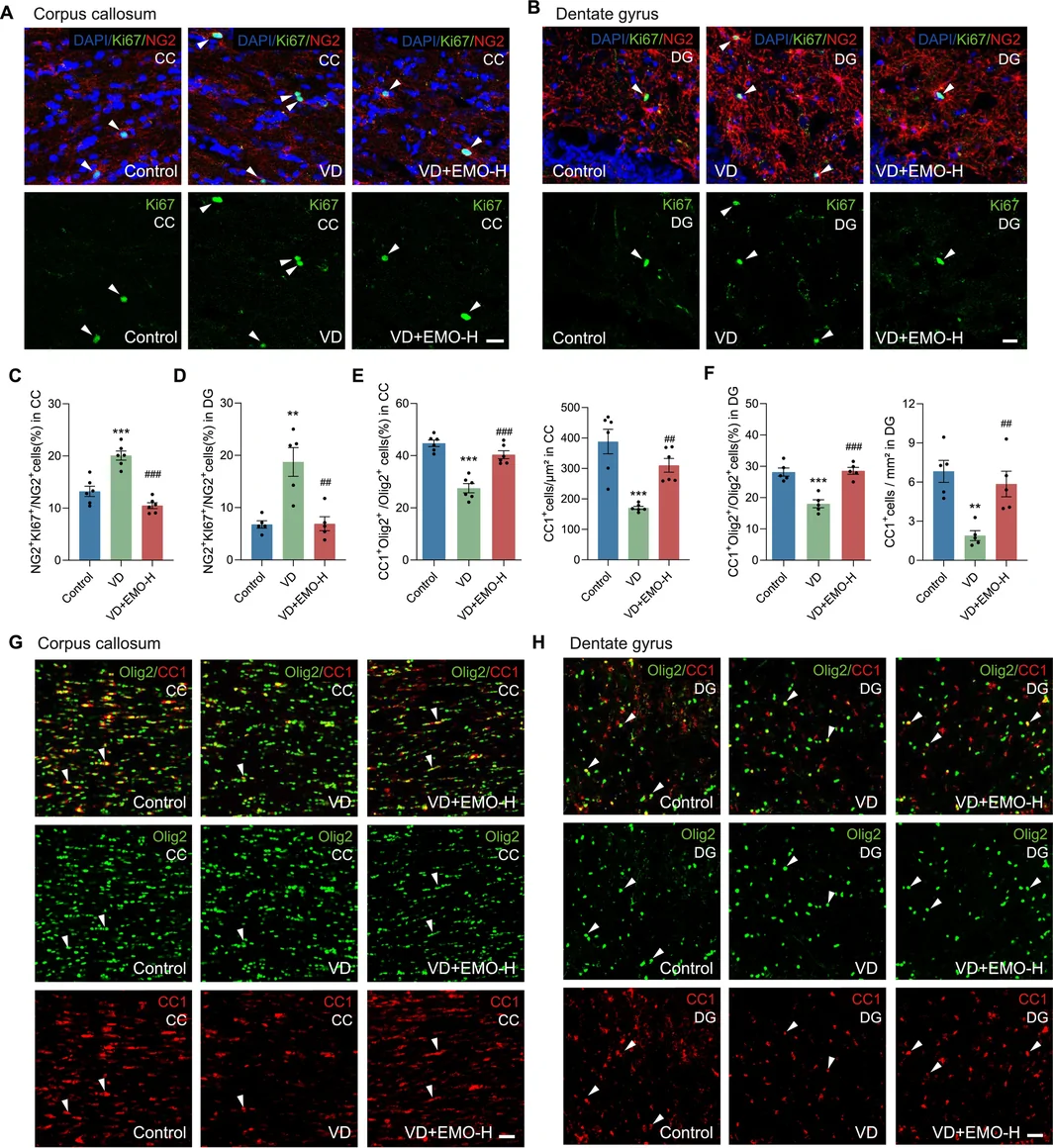
Emodin promotes OL lineage progression in the CC and hippocampal DG in VD rats. (A, B) Representative images of double‐staining for NG2 and Ki67 in the CC and DG. DAPI (nuclei, blue), NG2 (OPCs, red), Ki67^+^ (proliferating cells, green). White arrows indicate NG2^+^ Ki67^+^ proliferating OPCs. High‐magnification images were taken from the CC and DG separately. Scale bar: 20 μm. (C, D) The ratio of the number of NG2^+^Ki67^+^cells to that of NG2^+^ cells (%) in the CC and the DG. (E, F) The ratio of the number of Olig2^+^CC1^+^ cells to that of Olig2^+^ cells (%) and average number of CC1^+^ cells per mm^2^ in the CC and DG regions. (G, H) Representative images of double‐staining for Olig2 and CC1 in the CC and DG of the hippocampus. DAPI (nuclei, blue), Olig2 (oligodendrocyte lineage cells, green), CC1 (mature OLs, red). White arrows indicate Olig2^+^ CC1^+^ mature OLs. (**p < 0.01, ***p < 0.001 vs. Control; ##*p* < 0.01, ###*p* < 0.001 vs. VD. Data are represented as mean ± SEM, *n* = 5–6 rats per group, 3 brain slices per animal; CC, corpus callosum; DG, dentate gyrus).

The hippocampal dentate gyrus (DG) was selected for fluorescence assessment of oligodendrocyte lineage cells, as it exhibits a higher abundance of OLs than the CA1 [29, 30]. Notably, these two subfields are functionally linked via the trisynaptic circuit, suggesting that OL dysfunction in the DG could compromise CA1 neuronal integrity [31]. To determine whether emodin's pro‐remyelination effect is mediated by regulating OL function, we assessed OPC proliferation by co‐staining for NG2 (OPC marker) and Ki67 (proliferation marker [32]) both in the CC and DG. As shown in Figure 3A–D, VD model rats demonstrated a higher proportion of NG2^+^Ki67^+^ cells than controls, suggesting elevated OPC proliferation response. Following high‐dose emodin treatment, this increase was significantly reduced both in CC and DG.

To evaluate the effect of emodin on OL maturation, brain sections were co‐stained with Olig2, labeling entire oligodendroglial cells [33], and CC1, a specific marker for mature OLs [34]. In VD model rats, both the proportion and density of CC1^+^Olig2^+^ mature OLs were markedly reduced within the CC and DG compared to controls (Figure 3E–H). Conversely, high‐dose emodin treatment effectively reversed this reduction, suggesting that emodin promotes the maturation of OLs.

### 3.4 Emodin Inhibits the Activation of RAGE/ROCK1 Signaling Pathway in Response to VD

**FIGURE 4:**
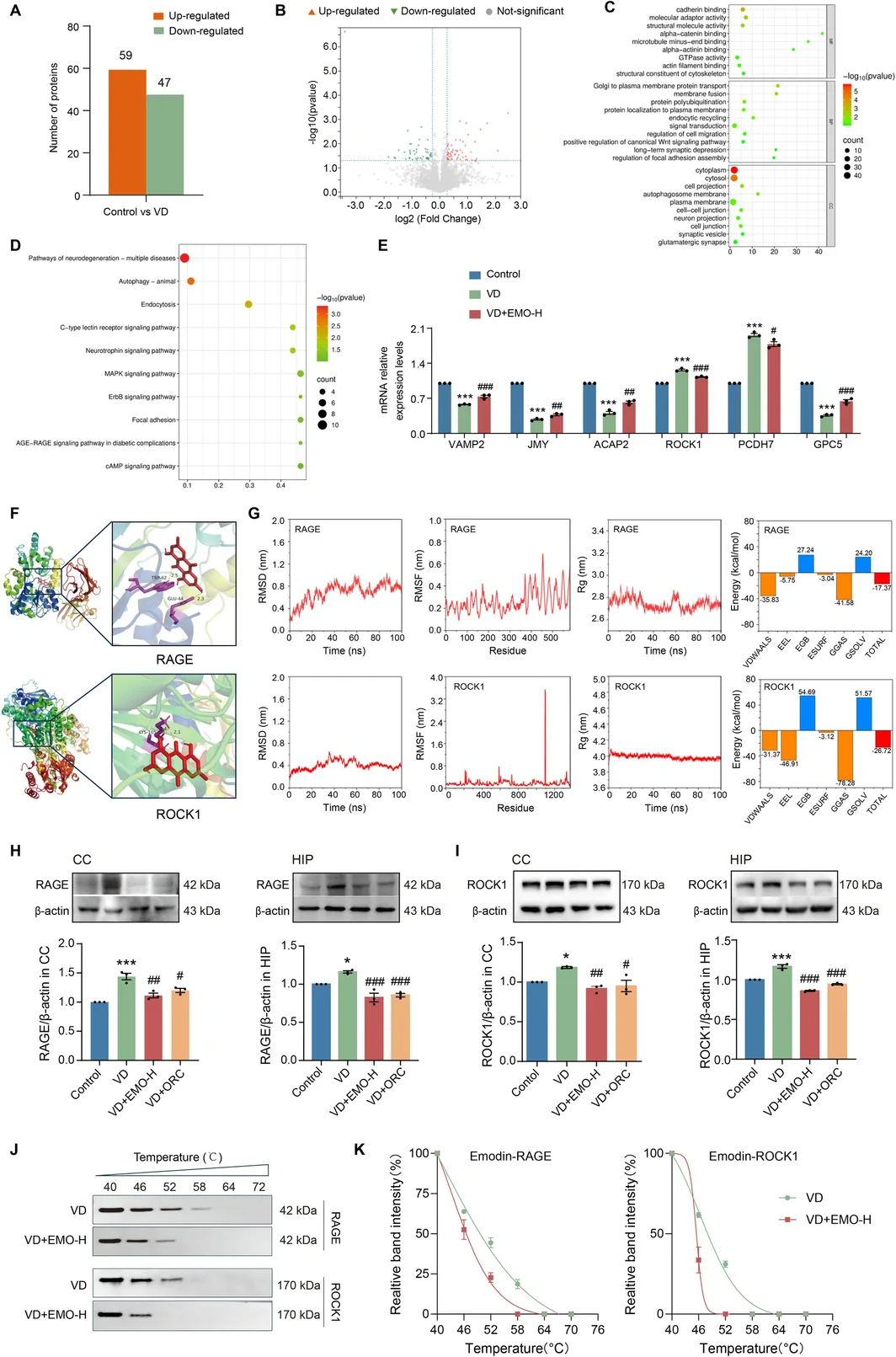
Integrated proteomics and network pharmacology identify RAGE/ROCK1 as the target of emodin in VD rats. (A) Proteins differentially expressed between the control and VD groups. (B) Volcano plot of control group versus VD group. (C) GO analysis of differentially expressed proteins (Control vs. VD; BP, biological process; CC, cellular component; MF, molecular function). (D) KEGG enrichment analysis of differentially expressed proteins (Control vs. VD). (E) mRNA relative expression levels of VAMP2, JMY, ACAP2, ROCK1, PCDH7, and GPC5. (F) Docking models of emodin binding to target proteins RAGE and ROCK1. (G) Molecular dynamics simulations of emodin‐RAGE and emodin‐ROCK1. (H, I) Western blot analysis of RAGE and ROCK1 expression in the CC and hippocampus. Data are normalized to β‐Actin protein expression. (J) Representative western blot images of RAGE and ROCK1 from CETSA. (K) Quantified melting curves of RAGE and ROCK1 (*p < 0.05, ***p < 0.001 vs. Control; #*p* < 0.05, ##*p* < 0.01, ###*p* < 0.001 vs. VD; data are represented as mean ± SEM, *n* = 3. CC, corpus callosum; Energy, binding free energy; HIP, hippocampus; Rg, radius of gyration; RMSD, root mean square deviation; RMSF, root mean square fluctuation).

To explore the mechanisms underlying emodin‐mediated myelin repair in VD, hippocampal tissues from VD and control rats were subjected to proteomic analysis. After excluding undetected proteins, 106 differentially expressed proteins were identified in the VD group, consisting of 47 downregulated and 59 upregulated targets (Figure 4A), as visualized in a volcano plot (Figure 4B). Given the importance of OL differentiation and myelination in CNS function and their disruption in neurological diseases [35], GO and KEGG enrichment analyses were conducted on these differentially expressed proteins to elucidate key regulatory pathways. As illustrated in Figure 4C, GO analysis indicated enrichment in biological processes such as focal adhesion assembly and signal transduction; cellular components including plasma membrane and cell–cell junction; and molecular functions like actin filament binding and structural constituent of cytoskeleton. KEGG analysis further demonstrated prominent enrichment in neurodegeneration, endocytosis, MAPK/ErbB, and focal adhesion pathways (Figure 4D). Given the close association of these pathways with OL biology and related neurological disorders [36, 37], six candidate proteins (VAMP2, JMY, ACAP2, GPC5, ROCK1 and PCDH7) were selected due to their co‐enrichment in these functional pathways and marked dysregulation in VD. qPCR analysis demonstrated that emodin administration successfully reversed the VD‐induced reduction in VAMP2, JMY, ACAP2, GPC5 mRNA levels, while also mitigating the upregulation of ROCK1 and PCDH7 (Figures 4E and S3).

RAGE and ROCK1 were prioritized based on KEGG enrichment, which showed the AGE‐RAGE pathway as a significantly enriched route in VD, where RAGE acts as the receptor and ROCK1 as a downstream mediator. Given the critical role of ROCK signaling in cytoskeletal reorganization, a process essential for OL differentiation and myelination [38, 39], we focused on ROCK1 and its upstream regulators, including RAF, NOTCH1, SIRT1, RhoA, and RAGE [40, 41, 42, 43]. Molecular docking indicated that emodin forms stable hydrogen‐bonding interactions with key residues across all six targets (Table 2, Figures 4F and S4). Notably, emodin exhibited strong binding affinity to RAGE and ROCK1, prompting further molecular dynamics simulations of RAGE‐emodin and ROCK1‐emodin complexes (Figure 4G). RMSD analysis showed both complexes stabilized following initial conformational adjustments (RAGE: 65–100 ns; ROCK1: 60–100 ns). RMSF analysis indicated localized flexibility in specific regions, while the radius of gyration confirmed structural compactness. The MM‐GBSA values for RAGE (−17.37) and ROCK1 (−26.72 kcal/mol) are relative estimates, within the expected range for this method (entropy excluded, solvation accounted) and comparable to reported emodin data [44], supporting favorable predicted binding interactions (Figure 4G).

**TABLE 2:** Molecular docking scores for Emodin with the associated proteins.

Target	PDB IDs	Docking energy (KJ/mol)
RAF	3IQU	−6.8
NOTCH1	2F8Y	−6.8
SIRT1	4KXQ	−7.2
RHOA	5KUT	−8.6
RAGE	3O3U	−8.8
ROCK1	7 S25	−9.3

To verify the effect of emodin on RAGE/ROCK1 signaling pathway, we assessed RAGE and ROCK1 expression. Compared with controls, the VD rats exhibited a considerable increase in RAGE and ROCK1 levels in both the CC and the hippocampus (Figure 4H,I). This elevation was reversed by high‐dose emodin and oxiracetam. These findings indicate that emodin suppresses RAGE and ROCK1 expression in VD rats, hence preventing the activation of the RAGE/ROCK1 pathway. Furthermore, CETSA performed on CC tissues from emodin‐treated rats showed reduced thermostability of RAGE and ROCK1 (Figure 4J,K), compared to the VD group. Although ligand binding often stabilizes proteins, CETSA can also detect compound‐induced thermal destabilization, as recently reported [45]. This leftward shift, together with our molecular docking and molecular dynamics simulations data, supports the notion that emodin directly engages with RAGE and ROCK1 in vivo.

### 3.5 Inhibition of RAGE/ROCK1 Signaling Pathway Promotes Remyelination and Cognitive Recovery in VD Rats

**FIGURE 5:**
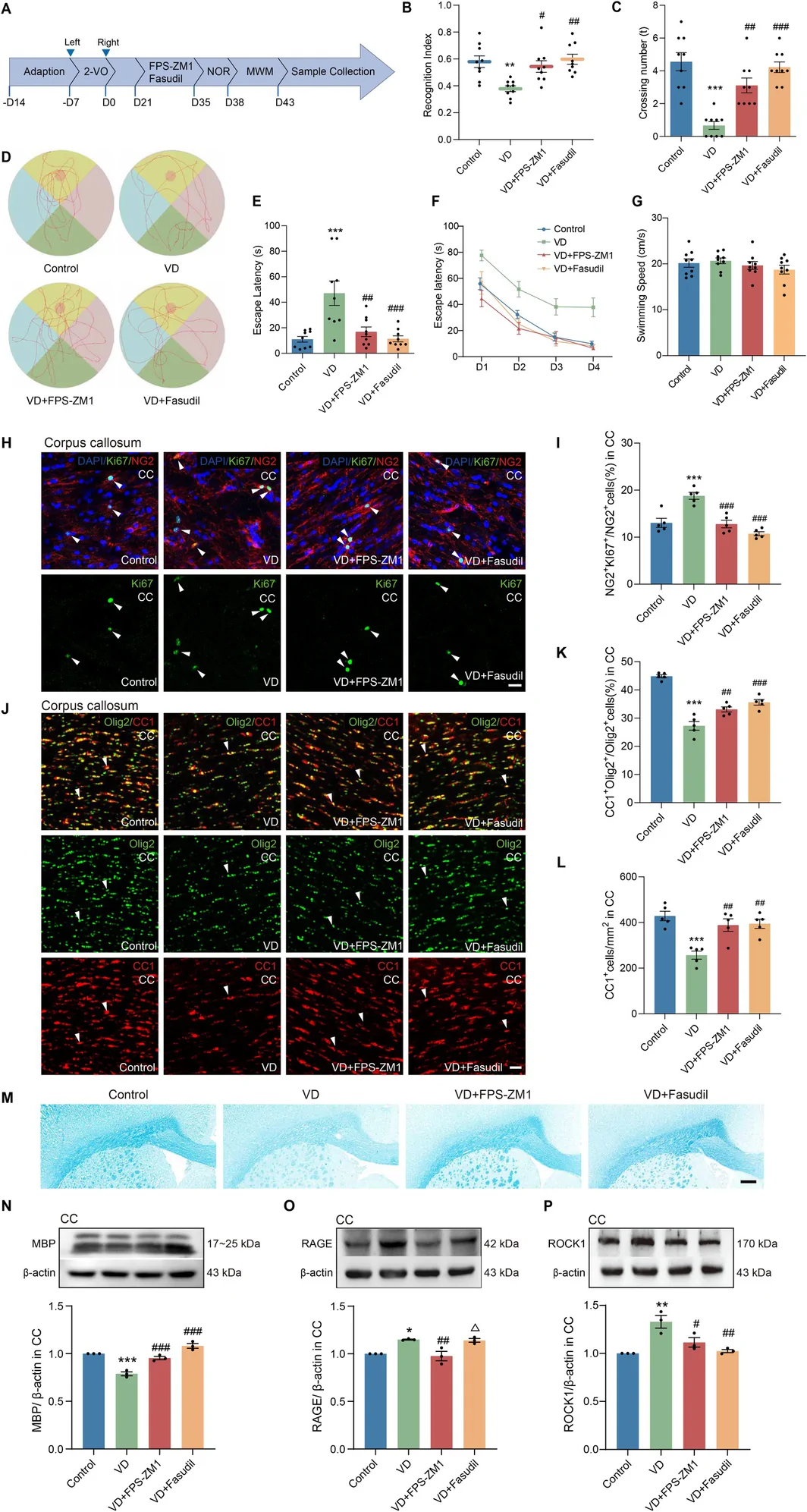
Inhibition of RAGE/ROCK1 signaling improves cognitive function, reduces OPC proliferative response, promotes OL maturation, and facilitates myelin repair in the CC of VD rats. (A) Schematic of experimental timeline. (B) NOR cognitive index in each group of rats (*n* = 9). (C) Number of platform crossings in the MWM (*n* = 9). (D) Representative search trajectories of each group during the MWM. (E) Escape latency, (F) average escape latency for the first 4 training days, and (G) swimming speed in the MWM (*n* = 9 per group). (H) Representative immunofluorescence images of NG2^+^Ki67^+^ proliferating OPCs (white arrows) in the CC. Scale bar: 20 μm. (I) Percentage of NG2^+^Ki67^+^/ NG2^+^cells in the CC region of each group. *n* = 5 rats per group, 3 brain slices per animal. High‐magnification images were taken from the CC. (J) Immunofluorescence images of CC1^+^Olig2^+^ mature OLs (white arrows) in the CC. Scale bar: 20 μm. (K) Percentage of CC1^+^Olig2^+^/Olig2^+^ cells, and (L) density of CC1^+^ cells in the CC. *n* = 5 rats per group, 3 brain slices per animal. (M) LFB staining of myelin in the CC. Scale bar: 200 μm. (N–P) Western blot analysis of MBP, RAGE, and ROCK1 expression in the CC (*n* = 3) (*p < 0.05, **p < 0.01, ***p < 0.001 vs. Control; #*p* < 0.05, ##*p* < 0.01, ###*p* < 0.001 vs. VD; Δ*p* < 0.05 vs. VD + FPS‐ZM1; data are represented as mean ± SEM).

To elucidate whether emodin improves cognitive function by blocking the RAGE/ROCK1 signaling pathway, we next assessed its effects in VD rats. On day 21 post‐modeling, VD rats received the RAGE inhibitor FPS‐ZM1 or the pan‐ROCK inhibitor Fasudil for 14 days, followed by NOR test and MWM (Figure 5A). Compared with controls, VD rats showed a reduced cognitive index, prolonged escape latency, and fewer platform crossings (Figure 5B–F). Administration of either FPS‐ZM1 or Fasudil significantly reversed these deficits, improving the cognitive index and shortening escape latency, with the Fasudil group also showing a significant increase in platform crossings. Swimming speed was comparable across all groups (Figure 5G). We further examined the role of RAGE/ROCK1 in oligodendrocyte lineage dynamics. Consistent with prior findings, VD stimulated OPC proliferation (NG2^+^Ki67^+^ cells) in the CC, which was effectively suppressed by FPS‐ZM1 and Fasudil (Figure 5H,I). Moreover, the density and proportion of mature OLs (CC1^+^Olig2^+^) were significantly reduced in VD rats but were restored following FPS‐ZM1 or Fasudil treatment (Figure 5J–L). Additionally, assessment of myelin integrity via LFB staining and MBP expression revealed severe impairment in the VD group, which was significantly rescued by both FPS‐ZM1 and Fasudil treatments, as evidenced by restored MBP levels and myelin structure (Figure 5M,N). Furthermore, RAGE and ROCK1 expressions were considerably increased in the VD rats, indicating pathway activation (Figure 5O,P). FPS‐ZM1 intervention attenuated RAGE and its downstream effector ROCK1 expression, while Fasudil reduced ROCK1 expression without affecting RAGE expression. Elevated RAGE expression was observed in the Fasudil group compared to the FPS‐ZM1 group, supporting that RAGE acts upstream of ROCK1 in this pathway.

### 3.6 ROCK1 Activation Mitigates Emodin's Protective Effects

**FIGURE 6:**
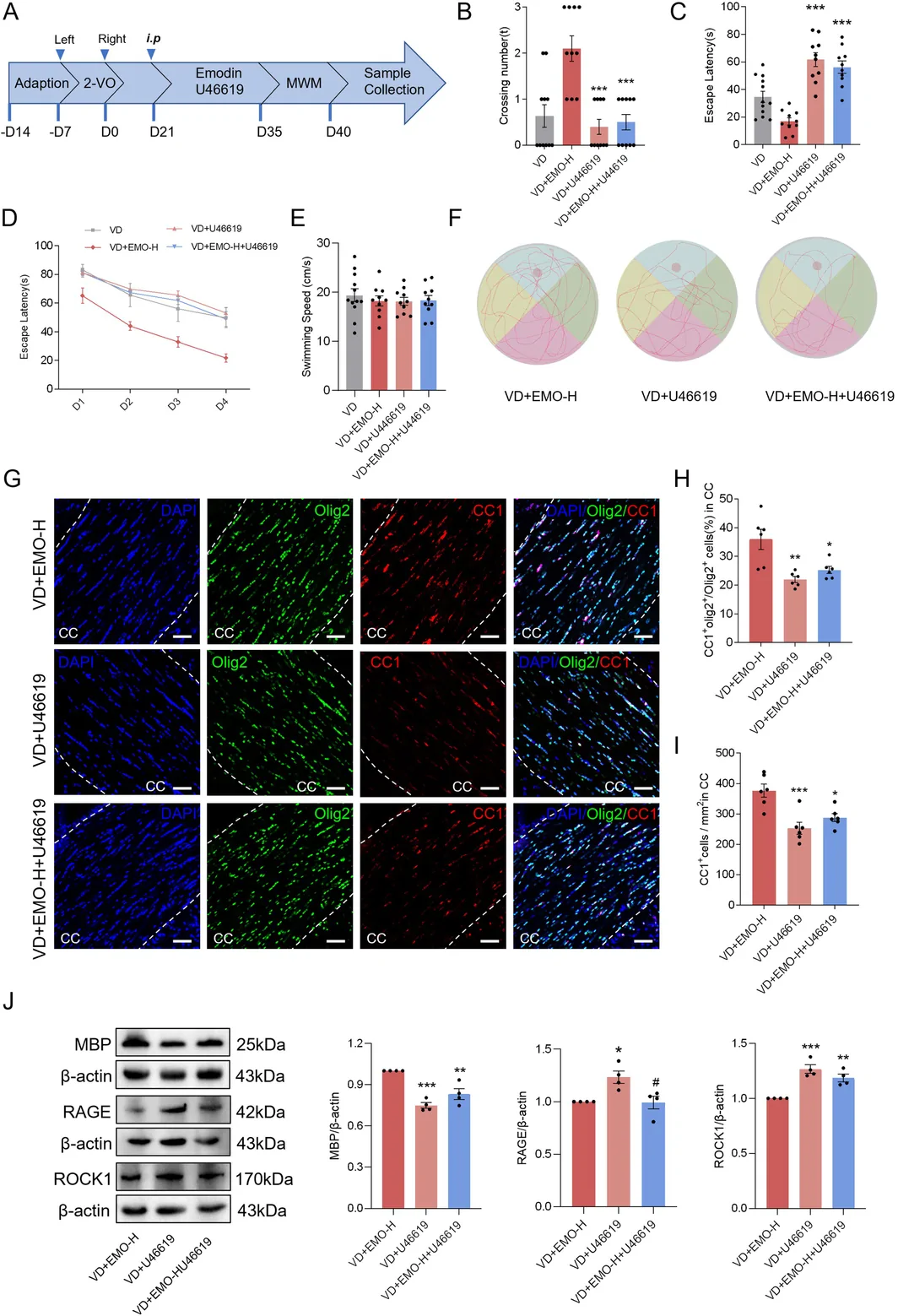
ROCK1 activation counteracts emodin‐induced cognitive and myelin protection in VD rats. (A) Schematic of experimental timeline. (B) Number of platform crossings, (C) escape latency, (D) average escape latency for the first 4 training days, and (E) swimming speed in the MWM (*n* = 10 per group). Data for the VD group were obtained from Figure 1 (*n* = 12 per group). (F) Representative search trajectories of each group in the MWM. (G) Immunofluorescence images of CC1^+^Olig2^+^ mature OLs in the CC. White dashed lines denote the CC borders. Scale bar: 50 μm. (H) Percentage of CC1^+^Olig2^+^/Olig2^+^ cells, and (I) density of CC1^+^ cells in the CC (*n* = 6 rats per group, 3 brain slices per animal). (J) Western blot analysis of MBP, RAGE, and ROCK1 expression in the CC (*n* = 4 rats per group) (*p < 0.05, **p < 0.01, ***p < 0.001, ****p < 0.0001 vs. VD + EMO‐H; #*p* < 0.05 vs. VD + U46619; CC, corpus callosum; data are represented as mean ± SEM).

Having established that RAGE/ROCK1 mimics emodin's effect, we next tested whether forced activation of this pathway would abolish emodin's protection. Co‐administration of the RhoA/ROCK agonist U46619 with emodin for 14 days from day 21 post‐modeling significantly impaired MWM performance versus the emodin‐alone group and was comparable to U46619 alone (Figure 6A–F).

Furthermore, the density and proportion of mature OLs, as well as MBP expression were lower in the combination group than in the emodin‐alone group, and were comparable to those in the U46619‐alone group (Figure 6G–J). U46619 acted through RhoA to upregulate ROCK1 [46]. In the presence of this agonist, emodin maintained its ability to suppress RAGE, without affecting ROCK1 elevation (Figure 6J). Consistently, MBP in co‐treated rats remained significantly lower than in emodin‐alone animals, revealing that ROCK1 activation overcomes emodin‐induced myelin repair. Together, these results indicated that ROCK1 inhibition is functionally required for emodin‐mediated cognitive and remyelinating effects in VD rats (Figure 6J).

## 4 Discussion

### 4.1 Emodin Promotes Myelin Repairment in VD Rats

VD, the second most prevalent dementia, is characterized by WMI induced by chronic cerebral hypoperfusion [19]. Given that remyelination relies on OPC differentiation and maturation [47], promoting myelin repair represents a key therapeutic strategy for VD. Emodin, a natural organic compound with well‐documented neuroprotective properties [48], has been reported to attenuate demyelination in CNS disorders [16, 17]. However, its effects on VD‐associated WMI have not been explored.

To investigate emodin's impact on OL function, WMI, and its associated molecular processes, we developed a well‐established 2‐VO rat model, which is widely used to mimic clinical VD‐related cognitive impairment and white matter damage [23]. Although staged occlusion lowered surgical mortality, it still produced chronic hypoperfusion and white matter lesions [49, 50]. Our results revealed that VD rats showed cognitive impairment alongside pathological and morphological alterations consistent with VD. It is worth noting that emodin treatment markedly improved cognitive function in VD rats, highlighting its neuroprotective role against 2‐VO‐induced cognitive decline. Electron microscopy and Cleave‐Caspase3 immunofluorescence further demonstrated that emodin treatment partially reversed the extensive damage to myelin and nerve fibers across multiple brain regions. Taken together, emodin emerges as a promising candidate for myelin repair in VD, with preliminary safety confirmed by liver and kidney toxicity tests.

### 4.2 Emodin Regulates the OL Lineage in VD Rats

Within the CNS, OLs are primarily responsible for forming myelin. Following demyelination, OLs must undergo a series of processes, progressing from OPCs to mature OLs that involve proliferation, morphological changes, and the expression of myelin protein markers. Since myelin impairment is a central mechanism in VD [51], therapeutic strategies aimed at preserving OLs, enhancing their differentiation, maturation, and facilitating remyelination are crucial for functional recovery. For instance, paeoniflorin can reverse WMI by reducing oxidative stress and inflammation, coupled with an increase in myelin‐binding protein expression [52]. Likewise, tretinoin alleviates CCH‐induced WMI by directly protecting OLs from apoptosis and indirectly suppressing microglia‐driven inflammation [53]. In this study, emodin similarly alleviated demyelination and apoptosis in the CC, suggesting its potential to support remyelination via modulating OL activity. Supporting earlier observations on OL‐mediated repair [54], we found that emodin restored disrupted OL homeostasis in VD rats. It reduced the elevated OPC proliferative response and spurred their differentiation into mature, myelinating OLs, indicated by heightened MBP and MOG. Newly formed OLs thus likely remyelinate impaired axons, providing a substrate for cognitive and functional restoration.

### 4.3 Emodin Likely Functions Through Inhibiting RAGE/ROCK1 Pathway to Exhibit the Protection

To further explore the mechanism through which emodin influences oligodendrocyte dynamics, proteomic screening followed by qPCR validation identified six OL‐related genes as potential targets. The RAGE/ROCK1 axis was selected for validation based on its proteomic enrichment and established roles in oligodendrogenesis and myelin repair [55]. Subsequent molecular docking, dynamics simulations, and CETSA analysis indicated that emodin binds with high affinity to the receptors for RAGE and ROCK1, suggesting these proteins may be key to its mechanism of action.

The migration and differentiation of OPCs are governed by cytoskeletal dynamics [56], a process potentially regulated by RAGE/ROCK1 signaling based on their respective roles in cell motility [57], and actin integrity [58]. RAGE activation by ligands such as AGEs or S100 proteins initiates pro‐inflammatory and pro‐oxidative signaling [59], and its overexpression disrupts oligodendrocyte lineage progression and myelination in neurological disorders [60, 61, 62, 63]. As a key downstream effector of Rho GTPases, ROCK1 is a pivotal regulator of cytoskeletal reorganization, inflammatory responses, and apoptosis [42]. Activation of ROCK1 worsens neuroinflammation and cognitive deficits [64], whereas its inhibition facilitates axonal regeneration and remyelination [65, 66]. Accordingly, the RAGE/ROCK1 signaling axis exerts a critical role in oligodendrocyte lineage progression and myelin repair. Our findings demonstrate that emodin acts by attenuating the activation of this pathway, as evidenced by its downregulation of both RAGE and ROCK1 protein levels. To functionally validate this mechanism, we blocked it using the pathway inhibitors. Either FPS‐ZM1 (a specific RAGE inhibitor) or Fasudil (a pan‐ROCK inhibitor) phenocopied emodin's effects. Although Fasudil lacks isoform selectivity, our proteomic analysis identified ROCK1, rather than ROCK2, as the differentially expressed isoform in VD rats. Definitive confirmation would require ROCK1‐selective siRNA or shRNA. Rescue experiments with U46619, a thromboxane A2 receptor agonist that activates RhoA/ROCK independently of RAGE, confirmed this mechanism. Co‐administration of U46619 with emodin reversed the cognitive improvement and myelin repair induced by emodin in VD rats, without affecting emodin‐mediated RAGE suppression. Meanwhile, the combination treatment did not lower ROCK1 expression, confirming U46619 agonism. This observation positions ROCK1 downstream of RAGE and underscores its role as the key effector of emodin's action. To test whether this pathway fully accounts for emodin's protective action, subsequent experiments should employ tissue‐specific ablation of RAGE or ROCK1.

Collectively, these results imply that emodin's inhibition of the RAGE/ROCK1 signaling pathway is crucial for ameliorating myelin damage. Although indirect effects from other cell types remain possible, CETSA confirms emodin binding to RAGE/ROCK1, and rescue with a ROCK1 agonist establishes that inhibition of this kinase is essential for the oligodendrocyte benefits. OPC‐specific or conditional knockout models are warranted to verify cell‐autonomous action.

### 4.4 Limitations of This Study

This study has several limitations. First, only male rats were utilized. Given estrogen's modulatory effects on WMI and remyelination [67, 68], it remains unclear whether these results extend to female animals. Second, the present work assessed OL lineage changes only at a single time point, which precludes distinguishing between reduced proliferative response and enhanced maturation. Future studies using primary OPC cultures are needed to directly address this question. Third, emodin was initiated 3 weeks post‐surgery as a restorative therapy without early prophylaxis, yet its efficacy in chronic VD supports its translational value. In addition, cardiovascular parameters were not monitored, although prior evidence indicates no cardiovascular or hepatorenal toxicity at emodin ≥ 40 mg/kg over 12–16 weeks [69, 70], and future inclusion of blood pressure monitoring would strengthen safety evaluation. Most importantly, this study was limited to morphological and structural analyses, and future functional exploration requires electrophysiology and other techniques.

In summary, emodin enhances cognitive function in VD rats by inhibiting the RAGE/ROCK1 signaling pathway, thereby modulating the OL lineage and promoting remyelination. These results provide substantial experimental support for the potential use of natural products in the treatment of VD and other demyelinating disorders.

## Author Contributions

**Xueqing Wang:** writing – original draft, validation, software, formal analysis, visualization, methodology, data curation, conceptualization. **Xiumei Dong:** validation, software, formal analysis, visualization, methodology, data curation. **Cunbao He:** methodology, visualization. **Junjie Zhang:** investigation. **Wenming Ban:** resources. **Guoqi Zhu:** supervision, writing – review and editing, project administration. **Jingji Wang:** conceptualization. **Yilan Zhen:** supervision, writing – review and editing, data curation, funding acquisition, conceptualization.

## Funding

This research was supported by grants from the Natural Science Foundation of the Anhui Education Department (2023AH050726) and the Talent Support Program of Anhui University of Traditional Chinese Medicine (DT2300000178) to Yilan Zhen. This research was also supported by the Basic‐Clinical Integration Program of Anhui University of Chinese Medicine (JCLCA2025003) for Prof. Guoqi Zhu, one of our co‐corresponding authors, and by the National Natural Science Foundation of China (82608237) for Dr. Yilan Zhen, our last corresponding author, which covered the publication fee.

## Ethics Statement

All animal experiments were performed in strict accordance with the guidelines of the Animal Ethics Committee at Anhui University of Chinese Medicine (Approval No. AHUCM‐rats‐2024163) and reported following the ARRIVE 2.0 guidelines.

## Conflicts of Interest

The authors declare no conflicts of interest.

## Supporting information


**Figure S1:** Emodin suppresses cell apoptosis in the corpus callosum region of 2‐VO‐induced VD rats. (A) Fluorescence images of cleaved Caspase 3 in the CC. DAPI (cell nucleus, blue), cleaved‐Casp3 (apoptotic cells, green), white dashed lines denote the CC borders. Scale bar: 100 μm. (B) Average pixel intensity of cleaved Caspase 3 in the CC (**p < 0.01 vs. Control; ##*p* < 0.01 vs. VD; data are represented as mean ± SEM, *n* = 3; CC, corpus callosum).
**Figure S2:** Correlation analysis between MBP expression and cognitive performance. (A) MBP expression negatively correlated with escape latency in the MWM (Pearson *r* = −0.9959, *p* < 0.0001, *n* = 6). (B) MBP expression positively correlated with recognition index in the NOR test (Pearson *r* = 0.9668, *p* = 0.0016, *n* = 6).
**Figure S3:** Melting curves (A) and amplification plots (B) for RT‐qPCR assays of Acap2, GPC5, JMY, PCDH7, Vamp2 and β‐actin.
**Figure S4:** Docking models of emodin binding to target proteins RAF (A), NOTCH1 (B), RhoA (C), and SIRT1 (D).

## Data Availability

Research data utilized for this study can be obtained from the corresponding author upon reasonable request.
