# Supplementary material for: Emodin Suppresses the RAGE/ROCK1 Pathway to Modulate the Oligodendrocyte Lineage and Ameliorate Demyelination in Vascular Dementia Rats
Source: CNS Neurosci Ther. 2026 Sep 16;32(9):e71154. doi: 10.1002/cns.71154 (PMC13579249; doi:10.1002/cns.71154)
Supplement: Supplementary file 1 [file CNS-32-e71154-s001.docx]

**Fig.S1 Emodin suppresses cell apoptosis in the corpus callosum region of 2‑VO‑induced VD rats.** (A) Fluorescence images of cleaved Caspase 3 in the CC. DAPI (cell nucleus, blue), cleaved-Casp3 (apoptotic cells, green), white dashed lines denote the CC borders. scale bar: 100 μm. (B) Average pixel intensity of cleaved Caspase 3 in the CC (^**^*p* < 0.01 vs. Control; ^##^*p* < 0.01 vs. VD; Data are represented as mean ± SEM, n=3; CC: corpus callosum)

**Fig.S2 Correlation analysis between MBP expression and cognitive performance.** (A) MBP expression negatively correlated with escape latency in the MWM (Pearson r = -0.9959, p <0.0001, n = 6). (B) MBP expression positively correlated with recognition index in the NOR test (Pearson r =0.9668, p = 0.0016, n = 6).

**Fig. S3 Melting curves (A) and amplification plots (B) for RT‑qPCR assays of *Acap2, GPC5, JMY, PCDH7, Vamp2* and *β‑actin.***

**Fig. S4 Docking models of emodin binding to target proteins RAF (A), NOTCH1 (B), RhoA (C), and SIRT1 (D).**
